# Mechanical Fault Diagnosis of a High Voltage Circuit Breaker Based on High-Efficiency Time-Domain Feature Extraction with Entropy Features

**DOI:** 10.3390/e22040478

**Published:** 2020-04-22

**Authors:** Jiajin Qi, Xu Gao, Nantian Huang

**Affiliations:** 1 Hangzhou Power Supply Company of State Grid, Hangzhou 310009, China; 2 Department of Electrical Engineering, Northeast Electric Power University, Jilin 132012, China; gaoxu55555@126.com (X.G.); huangnantian@neepu.edu.cn (N.H.)

**Keywords:** high voltage circuit breaker, fault diagnosis, time-domain segmentation, entropy feature, LightGBM

## Abstract

The fault samples of high voltage circuit breakers are few, the vibration signals are complex, the existing research methods cannot extract the effective information in the features, and it is easy to overfit, slow training, and other problems. To improve the efficiency of feature extraction of a circuit breaker vibration signal and the accuracy of circuit breaker state recognition, a Light Gradient Boosting Machine (LightGBM) method based on time-domain feature extraction with multi-type entropy features for mechanical fault diagnosis of the high voltage circuit breaker is proposed. First, the original vibration signal of the high voltage circuit breaker is segmented in the time domain; then, 16 features including 5 kinds of entropy features are extracted directly from each part of the original signal after time-domain segmentation, and the original feature set is constructed. Second, the Split importance value of each feature is calculated, and the optimal feature subset is determined by the forward feature selection, taking the classification accuracy of LightGBM as the decision variable. After that, the LightGBM classifier is constructed based on the feature vector of the optimal feature subset, which can accurately distinguish the mechanical fault state of the high voltage circuit breaker. The experimental results show that the new method has the advantages of high efficiency of feature extraction and high accuracy of fault identification.

## 1. Introduction

High voltage circuit breakers (HVCBs) are widely used in the power system and have complex mechanical operation structures. Their operation state is directly related to the power supply reliability of the power grid [1]. At present, the main diagnosis basis of high voltage circuit breaker fault diagnosis research includes vibration signal, sound signal, image signal, travel time of moving contact, and current signal of the opening (closing) coil [2,3,4,5]. During the operation of the high voltage circuit breaker, it will cause the start, braking, and impact of a series of components. The change of the movement form of these components will cause different degrees of vibration. These vibration waves are transmitted to the sensor through the structural components of the high voltage circuit breaker. When the circuit breaker has different mechanical faults, the vibration signals received by the circuit breaker are different. Therefore, the analysis of vibration signals generated by HVCBs action can find a variety of mechanical faults, such as insufficient spring energy storage, loose screws, etc. [6,7].

The fluctuation range of the vibration signal of the circuit breaker is wide and irregular. The feature set used in the fault diagnosis has a high feature dimension. The signal processing mostly uses the time–frequency analysis method, and the algorithm has a high time and space complexity. Common methods include S-Transform (ST) [8], Wavelet Transform (WT) [9], Empirical Mode Decomposition (EMD) [10], Local Mean Decomposition (LMD) [11], and Variable Mode Decomposition (VMD) [12]. ST provides a large number of time–frequency features, but it has a large amount of computation and is easily affected by parameters such as the window width factor [13]. WT has a good ability of local feature expression, but it is difficult to select wavelet bases in practical application [14,15]. EMD has poor separation ability for the components with similar frequency, and there is modal aliasing [16]; VMD decomposes the interference signal into multiple layers and extracts features from each layer of signal, but it is easily affected by the number of decomposition layers *K* and penalty coefficient *P* [17]. In reference [18], the permutation entropy is used as the fitness function, and the immune Drosophila optimization algorithm is used to search the combination parameters of *K* and *P* in VMD. The time–frequency analysis method has many problems, such as large calculation, difficult parameter selection, easy loss of high-frequency information, and so on, which will affect the classification effect of feature extraction after signal processing. For the mechanical fault diagnosis of a high voltage circuit breaker, the direct extraction of time-domain features can better reflect the mechanical state of the operating mechanism. Therefore, based on the time-domain segmentation of the original signal, using entropy features to represent the signal features in a specific period, extracting features directly from each time-domain signal can effectively shorten the time of feature extraction and ensure the integrity of feature information, effectively improve the efficiency of feature extraction, and meet the requirements of low-cost terminal edge calculation.

At present, the common methods of state recognition for HVCBs include Neural Network (NNs) [19], Random Forest (RF) [20], Support Vector Machine (SVM) [21,22], XGBoost [23,24], etc. NNs have a strong self-learning ability and nonlinear pattern recognition ability, but its training speed is slow and it is easy to fall into local optimal solution [25]. RF generalization ability is good, but the training time is long and memory consumption is large [26]. SVM is not easy to fall into the local optimal solution and overlearning, but the training speed is slow and the parameter selection is difficult. In reference [27], Particle Swarm Optimization (PSO) is used to optimize SVM. PSO is used to select penalty parameter c and kernel parameter g in SVM with a Gaussian kernel, which can effectively diagnose the tension fault of rope, but it is not suitable for the fault diagnosis of high voltage circuit breakers in a small sample scenario. XGBoost is an algorithm under the Boosting framework, which has good noise resistance, little influence of fitting problems, and better generalization ability. In reference [28], the combination of Complementary Ensemble Empirical Mode Decomposition and Extreme Gradient Boosting has significantly improved classification performance in terms of sensitivity, specificity, and accuracy. LightGBM is an improvement based on Gradient Boosting Decision Tree (GBDT) and XGBoost to ensure high efficiency and accuracy and prevent overfitting [29,30].

Based on the above factors, a new method of HVCBs mechanical fault diagnosis is proposed. Firstly, to improve the efficiency of feature extraction and retain more complete feature information, the original vibration signal of HVCBs was directly segmented in the time domain, and then 16 features including 5 kinds of entropy features were extracted from each original signal after time-domain segmentation to build the original feature set; then, to improve the efficiency of feature extraction, the Split importance value of each feature was calculated. Through the feature selection of the preceding item, the optimal feature subset was determined; finally, the features belonging to the optimal feature subset were input into LightGBM to establish an efficient HVCBs fault diagnosis LightGBM classifier. In the process of LightGBM construction, the search time was reduced by building the lightweight features of the vibration signals of high voltage circuit breakers; the multi-threaded parallel histogram acceleration was adopted to normalize all features in buckets, reducing the calculation amount and memory consumption, and effectively improving the training efficiency; the Leaf-wise growth strategy was adopted, and the maximum depth limit was increased, to improve the HVCBs fault diagnosis effect. The effectiveness and advanced nature of the new method were verified by comparative experiments.

## 2. Feature Extraction Based on Time-Domain Segmentation

### 2.1. Signal Acquisition System

The object of signal acquisition and analysis is a LW9-72.5 type circuit breaker, which is a kind of high voltage alternating current SF6 circuit breaker with three ceramic columns; the rated voltage is 66 kV, the rated frequency is 50 Hz, and a CT20 spring operating mechanism is adopted. The piezoelectric acceleration sensor is used to detect the vibration signal, and the sensor is fixed on the mechanism box near the operating mechanism of the high voltage circuit breaker by bolts. NI9234 and NI9401 data acquisition cards are used to collect data. NI equipment and the related LabView software are only used for data acquisition [11,15,17]. Taking the time when the breaker receives the opening action command as the coordinate zero point, the total sampling time is 2900 ms, and the sampling rate is 25.6 ks/s. The starting point of the vibration signal recording under four mechanical states is the same as the time length of the acquisition signal. The vibration signals of high voltage circuit breakers in four different states during opening operation are measured respectively: normal state; iron core jamming; screw loosening; insufficient lubrication. The system starts to collect vibration signals from the time when the circuit breaker receives the opening command and carries out multiple opening operations under the same conditions. A total of 50 groups of measured vibration signals under four states are obtained.

### 2.2. Time-Domain Division Method and The Basis of the Original Signal

**Figure 1:**
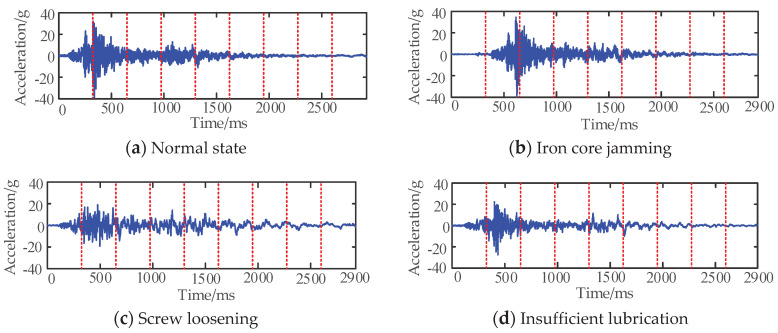
Time-domain division diagram of the original vibration signal.

To effectively extract the features of HVCBs original vibration signal in a specific period, the unified time scale was used to segment the original signal in the time domain. Different fault types were segmented in the same scale, and each segment of the segmented signal was extracted in the time domain. Under the normal working state of the HVCBs, the time from the opening command received by the operating mechanism of the circuit breaker to the peak value of the vibration signal amplitude of the circuit breaker was taken as a cycle, and the original vibration signal of the HVCBs was divided every other cycle, and finally, the original signal was divided into nine segments. Figure 1 is the schematic diagram of the original signal of the typical breaker fault and its time-domain division.

### 2.3. Feature Extraction based on Time-Domain Segmentation

In order to improve the efficiency of feature extraction and reduce the pressure of equipment cost, Time-Domain Segmentation (TDS) was directly applied to the vibration signal, and 16 kinds of time-domain features were extracted from each segmented segment for circuit breaker state identification and analysis. The 144-dimensional original feature set was constructed to evaluate the ability of feature combination and classification in different time domains.

It can avoid the loss of high-frequency information in time–frequency processing, ensure the integrity of feature information, and save signal processing time. It can be seen from the original vibration signal that, compared with the normal signal, the action of core jamming fault has a long delay time; the fluctuation amplitude of base screw loosening fault is small as a whole, and the attenuation speed is slow; the amplitude of poor lubrication fault of the crank arm is small, and the vibration time is longer. Therefore, in the time-domain segmentation, the difference between different types of fault signals can be more prominent, and then each segment of the segmented signal is used for feature extraction to form a feature vector to identify the state of HVCBs. In addition to the traditional time-domain features, the new method also adds five entropy features, which can better reflect the amplitude change degree and oscillation attenuation speed of signals in different periods after time-domain segmentation. Table 1 lists the calculation formulas and feature numbers of 16 features, respectively, where x(n) n=1,2,⋯,N is the amplitude corresponding to the nth sampling point, N is the total number of sampling points after time-domain segmentation, max is the function of taking the maximum value, min is the function of taking the minimum value, $p_{n}$ is the probability density of the nth sampling point, and α is the parameter for entropy calculation. The relevant calculation formula is shown in Table 1.

## 3. Feature Selection

Based on obtaining the original feature set, the forward feature selection was carried out to determine the optimal feature subset, and further reduce the calculation amount of features and the complexity of the classifier.

### 3.1. The Measurement Effect of Split Importance Value on Feature Classification Ability

**Figure 2:**
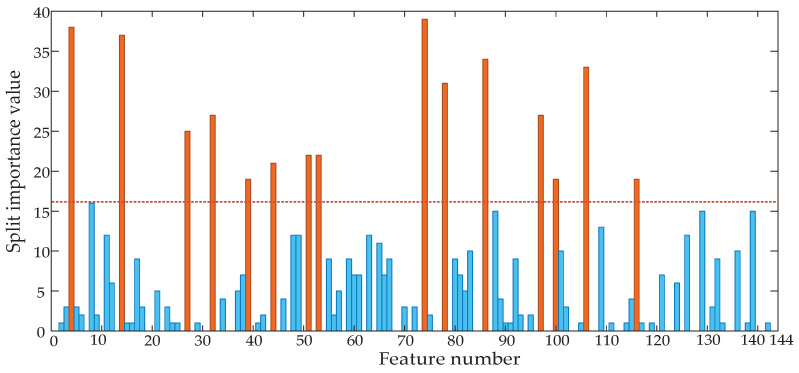
Split importance value of all features.

In the training process of LightGBM, the Split value of the feature represents the number of times the feature is used in the training, so the importance of the feature can be determined by calculating the Split value of the feature, and then the optimal feature subset can be constructed. First, we calculated the Split importance value of each feature of the original vibration signal after time-domain segmentation, and the calculation result is shown in Figure 2. After feature selection, the features belonging to the optimal feature subset were marked as orange, and the features not belonging to the optimal feature subset were marked as blue.

**Figure 3:**
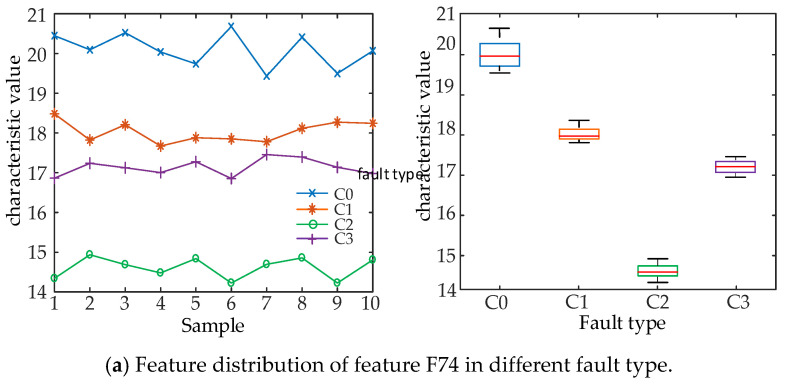

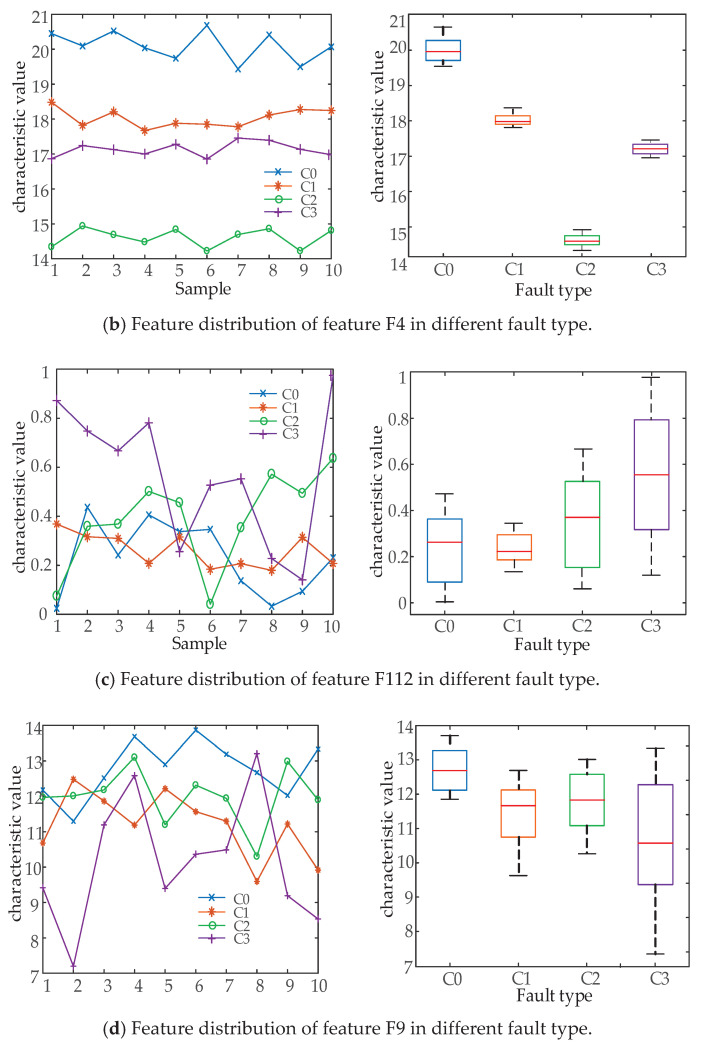
Distribution range of eigenvalues of high and low Split importance features in different fault types.

To verify the effectiveness of Split importance to measure the ability of feature classification, four features (F74, F4, F112, and F9) with the highest, higher, lower, and lowest importance values were selected in the original feature set Split importance ranking, F74 is the Waveform index of segment 2, F4 is the Peak value of segment 4, F112 is the Hartley entropy of segment 4, F9 is the Peak value of segment 9. We analyzed the distribution of eigenvalues of these four features in four different states. The equation of each feature can be found in Table 1. A total of 10 groups of data in each of four fault types were selected to calculate the eigenvalues, and the box line graph was constructed based on the distribution of the eigenvalues for display and analysis. The distribution of characteristics under different states of the circuit breaker is shown in Figure 3.

It can be seen from the analysis in Figure 3 that the eigenvalues of features F74 and F4 in four different fault types of HVCBs have obvious differences, with a small degree of cross between categories and good class separability. In contrast, the distribution of features F112 and F9 in four fault types have no obvious difference, with an obvious cross between categories and poor class separability. It shows that Split importance can effectively evaluate the time-domain feature classification ability of vibration signals.

### 3.2. Feature Selection based on Split Importance

**Figure 4:**
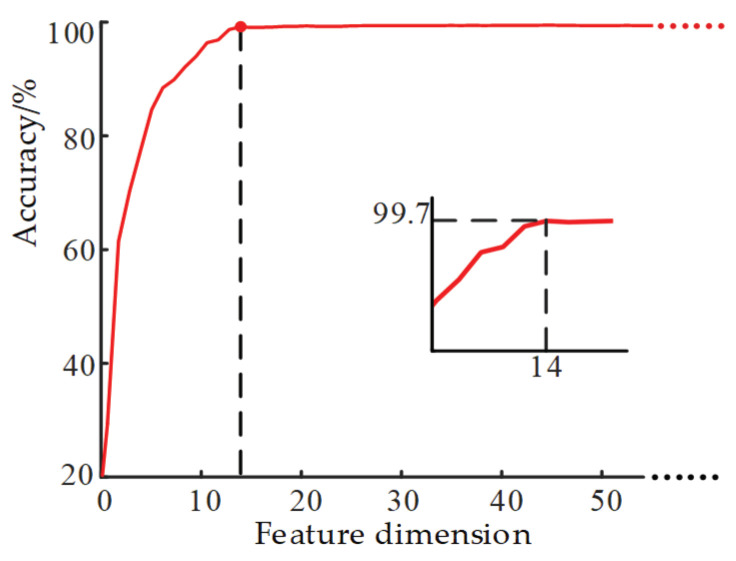
Accuracy of feature combination of different dimensions in forward feature selection.

The features were arranged in descending order according to Split importance, the forward feature selection was carried out, and the features are added to the feature subset in turn. For each feature added, the recognition accuracy of the classifier under the feature subset was calculated. This process was repeated until all features were added to the feature set, and then the optimal feature subset was determined according to the highest recognition accuracy. In the whole process, the recognition accuracy of the classifier changed as shown in Figure 4. When the feature dimension was 14 dimensions, the LightGBM classification accuracy reached the highest accuracy. The related characteristics of the optimal feature subset are described in Table 2. The optimal feature contained four types of entropy features, which reflect the ability of entropy features to represent signal characteristics in a specific period.

## 4. Construction of High-Efficiency Fault Diagnosis Classifier for High Voltage Circuit Breaker

LightGBM is a gradient promotion framework based on decision tree, which is an improved method of Gradient Boosting Decision Tree (GBDT) [31,32]. It solves the problems of overfitting and slow training in the field of HVCBs fault diagnosis with less training samples, enhances the robustness to noise, and ensures good evaluation accuracy and training speed. In the process of training LightGBM model for fault diagnosis of HVCBs, Gradient-based One-Side Sampling (GOSS) and Exclusive Feature Bundling (EFB) were used to preprocess the data, multi-thread parallel histogram was used to accelerate the training process, Leaf-wise growth strategy with depth limitation was used to avoid the overfitting risk caused by small fault samples, significantly improve the efficiency and accuracy of circuit breaker state identification, and enhance the generalization ability and anti-noise ability of the model.

### 4.1. Gradient Boosting Decision Tree

GBDT is an iterative decision tree algorithm based on the idea of Boosting iteration. In addition to the first decision tree generated by the original index, the goal of each iteration is to minimize the loss function value of the former, that is to say, the establishment of each decision tree is to reduce the residual of the previous model and reduce the residual to the gradient direction. The training process of GBDT is ladder-like, which needs to synthesize the results of all decision trees linearly to produce the final classification results. In the training process, the t tree is set as $f_{t}(x)$, and the $\theta_{t}$ tree is set as the parameter of the t tree. There are

(1) $$\left\{ \begin{array}{l} F_{0}(x)=0\\ F_{1}(x)=F_{0}(x;\theta_{0})+f_{1}(x;\theta_{1})\\ F_{2}(x)=F_{1}(x)+f_{2}(x;\theta_{2})\\ \;\vdots \\ F_{t}(x)=F_{t-1}(x)+f_{t}(x;\theta_{t}) \end{array}\right..$$

GBDT uses a decision tree to learn a mapping function from input space $X^{s}$ to gradient space G. Suppose there is a training set $\left\{x_{1},\ldots ,x_{n}\right\}$ with a data amount of n, where each $X_{i}$ is a vector of dimension s in space $X^{s}$. In each iteration, the negative gradient of the loss function is expressed as $\left\{g_{1},\ldots ,g_{n}\right\}$. The decision tree model is segmented at the feature with the largest information gain. The information gain is usually measured by the variance after Splitting and O is the data set in a fixed node of the decision tree. The variance gain of feature j at this node at partition point d is defined as

(2) $$V_{j|O}(d)=\frac{1}{n_{O}}(\frac{{\left(\sum_{\left\{x_{i}\in O:x_{ij}\leq d\right\}}g_{i}\right)}^{2}}{n_{l|O}^{j}(d)})+(\frac{{\left(\sum_{\left\{x_{i}\in O:x_{ij}>d\right\}}g_{i}\right)}^{2}}{n_{r|O}^{j}(d)}).$$

In the formula: $n_{O}=\sum I\left|x_{i}\in O\right|$, $n_{l|O}^{j}(d)=\sum I\left|x_{i}\in O:x_{ij}\leq d\right|$*,* $n_{r|O}^{j}(d)=\sum I\left|x_{i}\in O:x_{ij}>d\right|$. 

For feature j, the optimal partition point of the decision tree is $d_{j}^{*}=\arg\max_{d}V_{j}(d)$, and the maximum gain is $V_{j}(d_{j}^{*})$. Then the data is divided into left and right subtrees at point $d_{j}^{*}$ according to feature $j^{*}$.

### 4.2. Construction of Classifier Based on Time-Domain Characteristics of Vibration Signals of High Voltage Circuit Breakers

The traditional Boosting algorithm needs to scan all sample points of each feature to select the best segmentation point, which cannot meet the needs of HVCBs efficient fault diagnosis. LightGBM uses GOSS and EFB to preprocess the data and constructs the lightweight feature of the high voltage circuit breaker vibration signal, which can significantly reduce the search time and improve the training efficiency.

In the process of GOSS data sampling, only the data with a larger gradient was retained, and the overall distribution of data was not affected. First, the gradient values of data were sorted in descending order of absolute values, and the first a ∗ 100% data was selected, then b ∗ 100% data was randomly selected from the remaining smaller gradient data, and b ∗ 100% data was multiplied by a constant $\left(\frac{1-a}{b}\right)$ ∗ 100%. Finally, (a + b ∗ 100%) data was used to calculate the information gain. a is the sampling proportion of large gradient samples, and b is the sampling proportion of small gradient samples. Let a ∗ 100% be data subset *A*; let b ∗ 100% be data subset *B*. Finally, we calculate variance gain A∪B on union ${\hat{V}}_{j}(d)$. GOSS uses a small data set information gain to determine the segmentation point. The cost of calculating the information gain is greatly reduced, the training accuracy will not be lost too much, and the effect is better than the random sampling method.

(3) $${\tilde{V}}_{j}(d)=\frac{1}{n}(\frac{{\left(\sum_{x_{i}\in A_{l}}g_{i}+\frac{1-a}{b}\sum_{x_{i}\in B_{l}}g_{i}\right)}^{2}}{n_{l}^{j}(d)}+\frac{{\left(\sum_{x_{i}\in A_{\gamma }}g_{i}+\frac{1-a}{b}\sum_{x_{i}\in B_{\gamma }}g_{i}\right)}^{2}}{n_{r}^{j}(d)})$$

In the formula: $A_{l}=\left\{x_{i}\in A:x_{ij}\leq d\right\}$, $A_{r}=\left\{x_{i}\in A:x_{ij}>d\right\}$, $B_{l}=\left\{x_{i}\in B:x_{ij}\leq d\right\}$, $B_{r}=\left\{x_{i}\in B:x_{ij}>d\right\}$.

1. The feature dimension of the high voltage circuit breaker vibration signal is high. The number of features can be greatly reduced by combining sparse features with the EFB method. The fusion and binding of sparse features can be simplified as the problem of graph coloring. The specific steps are as follows: Let G = (V, E), take each row of the correlation matrix G as a feature, and then get V features. The mutually exclusive bundle is the vertex with the same color in the graph, the point in the graph is regarded as a feature, and the edge is regarded as the conflict between features. The optimal result of feature binding is determined by sorting according to the degree of mutual exclusion.
2. To ensure that the value of the original feature before binding can be recognized during feature binding, considering that the histogram algorithm after binding saves the continuous value as a discrete bucket, an offset constant is added to the feature value so that the value of different features can be divided into different buckets in the binding set.

For example, suppose that two features are in a feature bundle, the range of feature A is [0, 10], and the range of feature B is [0, 20]. Add an offset 10 to feature B, change it into [10, 30], and then merge it. Replace features A and B with a feature bundle [0, 30].

### 4.3. Multi-Thread Parallel Histogram Acceleration

**Figure 5:**
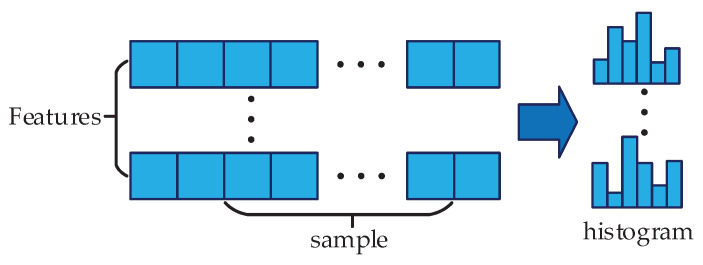
Histogram algorithm.

LightGBM uses histogram algorithm to normalize all features and divides the original continuous data into discrete *k* buckets, as shown in Figure 5. When traversing the data, the discrete value is used as the index, and the index value is accumulated in each bucket. After traversing the data once, the accumulation amount of each bucket can be obtained.

To improve the efficiency of fault diagnosis, a more efficient ergodic method should be used in the training process. The traditional Boosting algorithm needs to presort the features and save the sorted index value. Every time it traverses the segmentation point, it needs to calculate the Splitting gain, which is too expensive. In the new algorithm, the histogram of the leaf can be obtained by the difference between the histogram of its father node and that of its brother node, and the histogram of its brother leaf can be obtained only by traversing each bucket, which reduces the computation and memory consumption and improves the training efficiency effectively.

The fluctuation range of the vibration signal of the circuit breaker is wide and irregular. The data containing noise will fluctuate in a small range near the real value. When the model has a strong approximation ability to learn these fluctuations, it will cause the problem of overfitting. Using histogram algorithm, for each one-dimensional feature, the values in a certain range will be divided into the same bucket to obtain the same index, and thus can significantly improve the overfitting phenomenon caused by the vibration signal noise of the circuit breaker.

### 4.4. Leaf-Wise Growth Strategy With Depth Limitation

**Figure 6:**
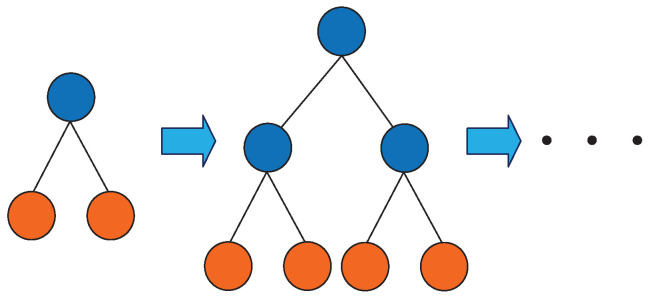
Level-wise growth strategy.

**Figure 7:**
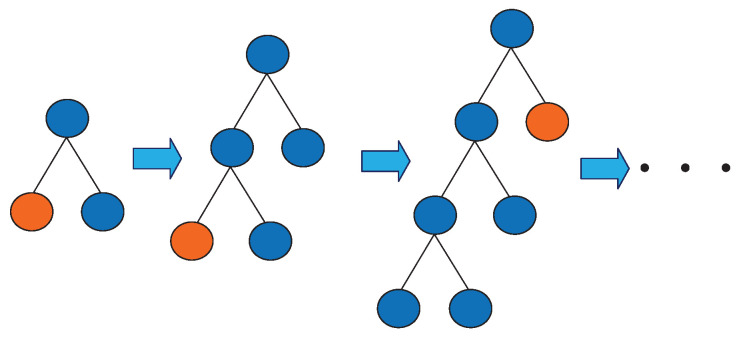
Leaf-wise growth strategy.

The vibration signal of the high voltage circuit breaker has a high time and space complexity. In the process of fault diagnosis, it should be used as short as possible to achieve a good classification effect. Moreover, overfitting should be avoided. In the Boosting method, the growth strategy of the decision tree directly affects the accuracy and efficiency of classification. The traditional Boosting method uses the Level-wise decision tree growth strategy as shown in Figure 6. It splits all leaves every time, but it wastes computing time and memory consumption to split the leaves with a low Splitting gain. LightGBM uses the Leaf-wise strategy with depth limitation as shown in Figure 7. It searches all the current leaves each time to find the leaf with the largest Splitting gain for Splitting. Compared with Level-wise, Leaf-wise can effectively reduce the calculation time, reduce the error, and improve the accuracy under the same Splitting times. However, it is easy to build a deep decision tree with a Leaf-wise growth strategy, resulting in overfitting. Therefore, LightGBM adds a maximum depth limit on Leaf-wise to ensure high efficiency and precision while preventing overfitting.

## 5. Case Analysis of Fault Diagnosis

### 5.1. Process of Fault Diagnosis

**Figure 8:**
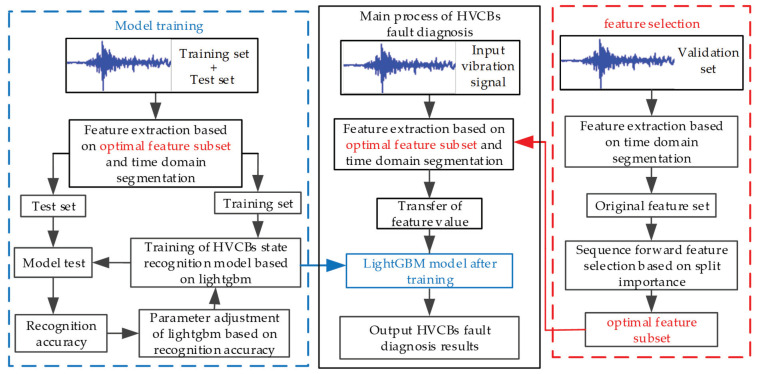
High voltage circuit breakers (HVCBs) fault diagnosis flow chart.

The new scheme mainly includes feature extraction, feature selection, and fault diagnosis. In the fault diagnosis of HVCBs, firstly, the vibration signal of the target HVCBs was collected, then the original vibration signal was extracted based on the time-domain segmentation and the optimal feature subset. Finally, the feature was input into the trained LightGBM model to realize the circuit breaker state recognition. The troubleshooting process is shown in Figure 8.

### 5.2. Efficiency Analysis of Feature Extraction based on Time-Domain Segmentation

**Figure 9:**
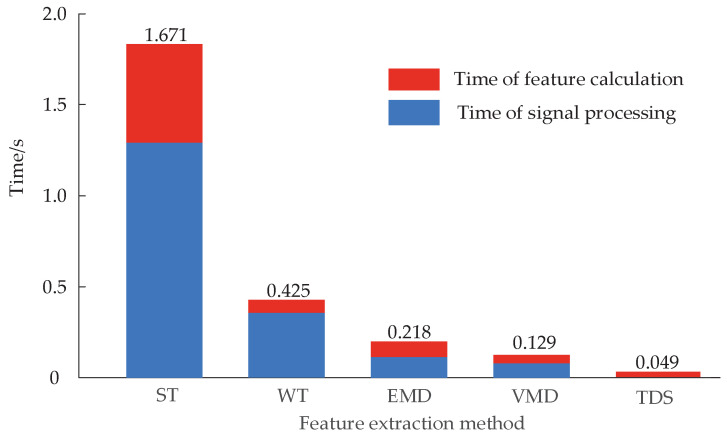
Feature extraction time of different methods.

In order to analyze the efficiency of the new feature extraction method based on time-domain segmentation compared with the traditional method, the feature extraction time required by the edge side for the same group of vibration signals under different feature extraction methods is shown in Figure 9. According to the analysis of Figure 9, compared with ST, WT, EMD, and VMD, the new method has no signal processing time, and the overall feature extraction time is far lower than each feature extraction method based on signal processing. Therefore, it is of great practical significance to simplify the process of feature extraction and improve the efficiency of feature extraction while ensuring high classification accuracy.

### 5.3. Analysis of Classification Effect of LightGBM 

To verify the classification accuracy of LightGBM, RF, SVM, GBDT, XGBoost, and LightGBM 5 classifiers were used to diagnose the fault types of HVCBs. In the experiment, the same time-domain segmentation method and the same feature extraction formula were used, and the forward feature selection method was used to determine the optimal feature subset of different classifiers according to the classification accuracy of different classifiers. In the process of feature selection, the classifier established under different feature subsets was optimized to minimize the classification error rate, and the best parameters of the classifier are determined by 10 fold cross-validation combined with Bayesian optimization [33]. The optimal feature dimensions and optimal accuracy of various classifiers are shown in Table 3. By comparison, it is found that under the premise of the highest accuracy, the dimension of the optimal feature subset corresponding to LightGBM is the smallest.

**Figure 10:**
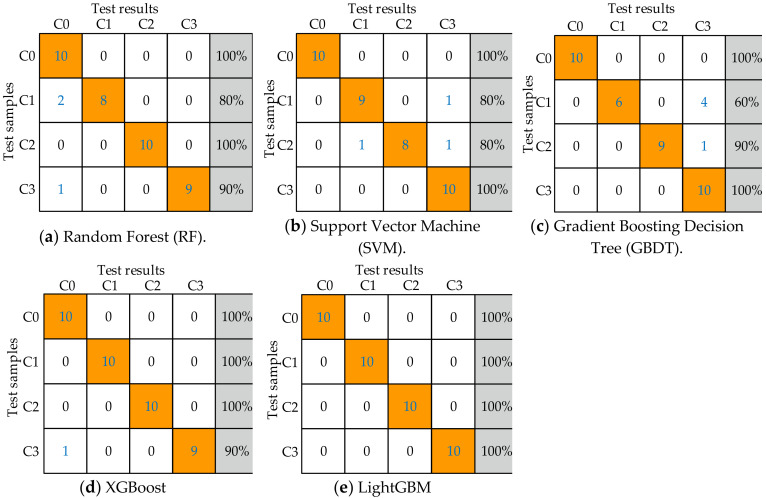
Classification results of five different classifiers.

According to the optimal classifier constructed in Table 3, another 10 sets of data were selected to verify the performance of the optimal classifier, and the classification results are shown in Figure 10. (RF) [20], (SVM)

From the analysis of Figure 10, it can be seen that RF has an average accuracy of 92.5% in identifying C1 and C3; SVM has an average accuracy of 90.0% in identifying C1 and C2; GBDT has an average accuracy of 87.5% in identifying C1 and C2; XGBoost has an average accuracy of 97.5% in identifying C3, and LightGBM has an accuracy of 100% in identifying four states.

High voltage circuit breakers cannot operate frequently because of its working characteristics, and thus it requires a high accuracy of state identification and overall efficiency of fault diagnosis. LightGBM adopts the data preprocessing method of GOSS and EFB and the multi-thread parallel histogram acceleration method to shorten the fault diagnosis time to the greatest extent and ensure the transmission efficiency of HVCBs vibration signal data. At the same time, it adopts the Leaf-wise growth strategy with depth limitation, which can effectively avoid overfitting caused by noise and has high classification accuracy. Therefore, LightGBM can improve the efficiency and accuracy of fault diagnosis and ensure the normal operation of the HVCBs.

### 5.4. Comparative Experiment of Circuit Breaker Fault Diagnosis based on Vibration Signal in the Noise Scene

To promote the application of related research results in practical projects, the new method will try to apply to the low-cost embedded system which is easy to introduce more noise signals in the process of data acquisition. To verify the diagnosis efficiency and classification accuracy of the new method under the noise environment, the vibration signal of the circuit breaker with a signal-to-noise ratio of 30 dB after adding white noise was taken as the experimental object. The new method and the method in [15,17,34] were used to carry out fault diagnosis, respectively, and the effectiveness and robustness of different methods were compared. Among them, literature [15,17] uses the time–frequency analysis method, without feature selection. Both the new method and the reference [34] adopt the feature extraction method based on time-domain segmentation, and both of them contain feature selection links, but the scale, type, and number of features in time-domain segmentation are also different. In the experiment, the data and results of feature extraction and classification in the experiment are shown in Table 4. It can be seen from Table 4 that the new method has a high utilization rate for entropy features, short feature extraction time, the highest classification accuracy, and the best noise resistance.

In the feature extraction of the vibration signal of the circuit breaker, the time–frequency analysis method of WT is adopted in reference [15], the signal decomposition time is long, the selection of the wavelet base is difficult, and the high-frequency information of the signal is easy to be lost. In reference [17], the time–frequency domain analysis method of VMD is easy to be affected by the number of decomposition layers *K* and penalty coefficient *P*, and the error of parameter setting will affect the final feature extraction effect. In reference [34], the time-domain features of the original signal are extracted directly, which improves the efficiency of feature extraction. However, the time-domain segmentation scale is narrow, the number of features in the original feature set is large, and the type of entropy features is small. The new method uses more types of entropy features in feature extraction, and the optimal feature set contains four entropy features. It can be seen that entropy features contribute a lot to fault diagnosis of circuit breakers, which promotes the application of relevant entropy features in fault diagnosis.

In the circuit breaker fault diagnosis, the OCSVM method is used in reference [15], which can only identify the fault and no-fault states of the circuit breaker, and has strong limitations. A multi-layer classifier is used in reference [17], which needs to be judged many times in the process of fault diagnosis and has poor anti-noise ability and low classification accuracy. OCSVM-RF-OCSVM used in reference [34] is a combination of two classifiers. The accuracy of fault diagnosis is higher than that of using OCSVM or RF alone. However, the classification boundary of OCSVM is relatively strict, and it is easy to recognize normal signals as fault signals in noisy environments (from the experimental results in Table 4, it can be seen that there are 2.5% normal noisy signals that are mistakenly recognized as fault signals), and in the combined pattern recognition method, the optimal feature subsets of OCSVM and RF are different, so it is difficult to determine them uniformly. The new method optimizes the LightGBM classifier with a Leaf-wise growth strategy with depth limitation, reduces the risk of overfitting caused by small fault samples, and uses the GOSS and EFB methods to preprocess the data, classifies the processed lightweight features, and improves the anti-noise ability significantly. Compared with other classification methods, LightGBM can correctly distinguish the state of the circuit breaker under the noise environment, which is of great significance to improve the operation reliability of the high voltage circuit breaker.

## 6. Conclusions

The new method mainly realizes the feature extraction of HVCBs original vibration signal and the accurate recognition of HVCBs state and shows the good effect of the new method in the diagnosis example through the contrast experiment. The advantages of this method are as follows:

- It can guarantee the integrity of the feature information, shorten the time of feature extraction, and the effect of feature classification is good;
- Based on the Split importance value, the forward feature selection is carried out to determine the optimal feature set, which effectively reduces the amount of feature calculation and the complexity of the classifier;
- LightGBM is introduced into the fault diagnosis of a high voltage circuit breaker, the data is preprocessed by the GOSS and EFB methods, the training is accelerated by the multi-threaded parallel histogram, and the Leaf-wise growth strategy with depth limitation is adopted to effectively avoid the overfitting risk caused by small fault samples, improve the efficiency and accuracy of circuit breaker state identification, as well as enhance the generalization ability and anti-noise ability of the classifier.

The new fault diagnosis method can identify the mechanical state of the high voltage circuit breaker accurately and efficiently and has a wide application prospect in the fault diagnosis of a high voltage circuit breaker. The new method presented in this paper is mainly based on the vibration signals. In the future, based on the existing research ideas, more abundant features, such as contact travel, timing, coil current, etc., will be introduced to further improve the accuracy of related fault diagnosis. At the same time, we will try to use a low-cost embedded system, combined with communication technology, to form a complete condition monitoring system and to promote the application of related research results in the actual industrial area.

## Figures and Tables

**Table 1:** Calculation formula of each feature.

Features	Formula	Feature Number	Features	Formula	Feature Number
Peak value	$F_{pv}=\max\left(\left|x(n)\right|\right)$	F1–F9	Waveform index	$F_{sf}=\frac{F_{rms}}{F_{av}}$	F73–F81
Mean value	$F_{mv}=\frac{1}{N}\sum_{n=1}^{N}x(n)$	F10–F18	Pulse index	$F_{if}=\frac{F_{pv}}{F_{av}}$	F82–F90
Standard deviation	$F_{std}=\sqrt{\frac{1}{N}\sum_{n=1}^{N}{\left(x(n)-F_{mv}\right)}^{2}}$	F19–F27	Clearance index	$F_{mf}=\frac{F_{pv}}{F_{sra}}$	F91–F99
Variance	$F_{tv}=\frac{1}{N}\sum_{n=1}^{N}{\left(x(n)-F_{mv}\right)}^{2}$	F28–F36	Collision entropy	$F_{ce}=-log\sum_{n=1}^{N}{p_{n}}^{\alpha }$	F100–F108
Skewness	$F_{sv}=\frac{1}{N}\sum_{n=1}^{N}{\left(\frac{x(n)-F_{mv}}{F_{std}}\right)}^{3}$	F37–F45	Hartley entropy	$F_{he}=log\sum_{n=1}^{N}{p_{n}}^{\alpha }$	F109–F117
Kurtosis	$F_{kv}=\frac{1}{N}{\sum_{n=1}^{N}\left(\frac{x(n)-F_{mv}}{F_{std}}\right)}^{4}$	F46–F54	Shannon entropy	$F_{se}=-\sum_{n=1}^{N}p_{n}\log p_{n}$	F118–F126
Square root amplitude	$F_{sta}={\left(\frac{1}{N}\sum_{n=1}^{N}\sqrt{\left|x(n)\right|}\right)}^{2}$	F55–F63	Tsallis entropy	$F_{te}=-\frac{1}{\alpha -1}\log(1-\sum_{n=1}^{N}{p_{n}}^{\alpha })$	F127–F135
Peak to peak value	$F_{ppv}=\max(x(n))-\min(x(n))$	F64–F72	Renyi entropy	$F_{re}=\frac{1}{1-\alpha }log\sum_{n=1}^{N}{p_{n}}^{\alpha }$	F136–F144

**Table 2:** The characteristics of the optimal feature subset.

Feature Number	Feature Description	Feature Number	Feature Description
F74	Waveform index of segment 2	F97	Clearance index of segment 7
F4	Peak value of segment 4	F27	Standard deviation of segment 9
F14	Mean value of segment 5	F51	Kurtosis of segment 6
F86	Pulse index of segment 5	F53	Kurtosis of segment 8
F106	Collision entropy of segment 7	F44	Skewness of segment 8
F129	Tsallis entropy of segment 3	F100	Collision entropy of segment 1
F33	Variance of segment 6	F116	Hartley entropy of segment 8

**Table 3:** Feature selection of different classifiers.

Classifier	Optimal Feature Dimension	Optimal Accuracy
RF	31	95.83%
SVM	24	95.00%
GBDT	19	93.33%
XGBoost	17	97.50%
LightGBM	14	99.17%

**Table 4:** Comparison of experimental data.

Method	Feature Extraction Method	Feature Dimension of Original Feature Set	Entropy Feature Type of Original Feature Set	Feature dimensions of Optimal Feature Set	Entropy Feature Number of Optimal Feature Set	Extraction Time	Classification Method	Classification Accuracy
NEW Method	TDS	144	5	14	4	0.049s	LightGBM	100.00%
Reference 15	WT	40	1	-	-	0.754s	OCSVM	92.50%
Reference 17	VMD	30	0	-	-	0.429s	Multi-Layer Classifier	96.25%
Reference 31	TDS	493	3	12	3	0.041s	OCSVM-RF-OCSVM	97.50%
